# The relationship between circulating metabolites and prostate hyperplasia: a Mendelian randomization study

**DOI:** 10.1007/s40520-023-02669-4

**Published:** 2024-01-28

**Authors:** Ze-chao Zhang, Shu-ping Huang, Ze-sen Lin, Yu Chen, Peng Jiang, Yu-wei Yang, Chang-jie Shang, Min Zhu, Hong-jun Gao

**Affiliations:** 1 Ruikang Hospital Affiliated to Guangxi University of Chinese Medicine, No. 10 Huadong Road, Xingning District, Nanning, 530000 People’s Republic of China; 2 The Second People’s Hospital of Zhaoqing, Zhaoqing, 526000 China; 3 Guangxi Center of Clinical Medicine for Kidney Diseases, Nanning, 530000 China

**Keywords:** Prostatic hyperplasia, Circulating metabolites, Mendelian randomization

## Abstract

**Background:**

Circulating metabolites (CM) play a pivotal role in our overall health, yet the current evidence concerning the involvement of diverse CM in benign prostatic hyperplasia (BPH) remains limited. Mendelian randomization (MR) offers a promising avenue to explore the potential impact of CM on BPH.

**Methods:**

In a forward MR analysis, a cohort of 249 circulating metabolites was employed as exposures to investigate their potential associations with BPH risk. Conversely, in a reverse MR analysis, BPH was employed as an exposure to assess its effects on CM.

**Results:**

The forward MR analysis discerned a linkage between six metabolites and BPH, with careful consideration to excluding heterogeneity and pleiotropy. Subsequently, the reverse MR analysis unveiled that nine metabolic compounds, mainly comprising phospholipids and triglycerides, potentially exhibit elevated levels in BPH patients.

**Conclusion:**

Bidirectional MR analysis furnishes genetic insight into the interplay between CM and BPH. The prominence of lipids and triglycerides emerges as significant factors intricately linked to BPH risk.

**Supplementary Information:**

The online version contains supplementary material available at 10.1007/s40520-023-02669-4.

## Introduction

Benign prostatic hyperplasia (BPH): Benign prostatic hyperplasia (BPH) represents the foremost benign ailment catalyzing voiding abnormalities in middle-aged and elderly males. The incidence of BPH surges substantially, reaching up to 50%, in men aged over 60 years [1]. BPH emerges as a quintessential ailment of the aging process. Notably, with the advancing male age, the incidence and prevalence of BPH escalate concomitantly. Despite a plethora of scholarly investigations, the exact etiological basis remains a subject of controversy. Various theories, spanning genetics, androgens, hormones, cytokines, chemokines, and the pivotal role of stem cells, have been posited as potential determinants driving disease progression. These factors are widely acknowledged as pivotal contributors fostering the advancement of the condition. Nevertheless, thus far, a consensus concerning the etiology of BPH remains elusive [2]. The escalating number of patients seeking treatment for BPH-associated lower urinary tract symptoms (LUTS), coupled with the exponential rise in healthcare expenditures, underscores the pressing need for efficacious therapeutic interventions [3]. Hence, a proactive exploration into the etiological underpinnings and influential factors governing BPH assumes paramount significance in the realm of its management. Metabolomics analysis revealed that the specific metabolites were related to the disease progression of BPH. Prostate diseases can cause metabolic abnormalities, thus clarifying the relationship between various metabolites and prostate diseases is helpful to identify benign and malignant prostate diseases, so as to reduce the excessive treatments and economic burden in society and improve the living quality of patients [4]. Notably, some studies have demonstrated that serum metabolites become potential biomarkers of BPH owing to their superior sensitivity and specificity in identifying BPH [5]. Therefore, exploring the relationship between metabolites and BPH is helpful for the diagnosis and treatment of BPH. Mendelian randomization provides a pathway to explore the bidirectional relationship between circulating metabolites (CM) and BPH from the perspective of genetic exposure.

Mendelian randomization (MR): MR constitutes an epidemiological approach that capitalizes on genetic variants as instrumental variables to serve as surrogates for the variables of interest, thereby facilitating the evaluation of the causal repercussions of exposures on specific outcomes[6]. The inherent random assignment of single nucleotide polymorphisms (SNPs) confers resistance against confounding influences. Importantly, genetic variations remain immune to the development of subsequent outcome traits, mitigating the specter of reverse causality bias [7]. Augmenting its utility, genetic instrumental variables encapsulate lifelong exposures, rendering MR an adept tool for scrutinizing age-associated phenomena. In the contemporary landscape, bolstered by high-throughput metabolomics, the concurrent measurement of numerous circulating metabolite levels has become feasible. Recent epochs have witnessed a plethora of genome-wide association studies (GWASs) delving into the nexus between circulating metabolic biomarkers and SNPs [8–10]. In the present study, leveraging the largest human genomic dataset available to date, we employ the bidirectional MR methodology to discern the causal nexus between circulating metabolites and the susceptibility to the prevalent BPH characteristic among the elderly demographic.

## Materials and methods

### Research design description

**Fig. 1:**
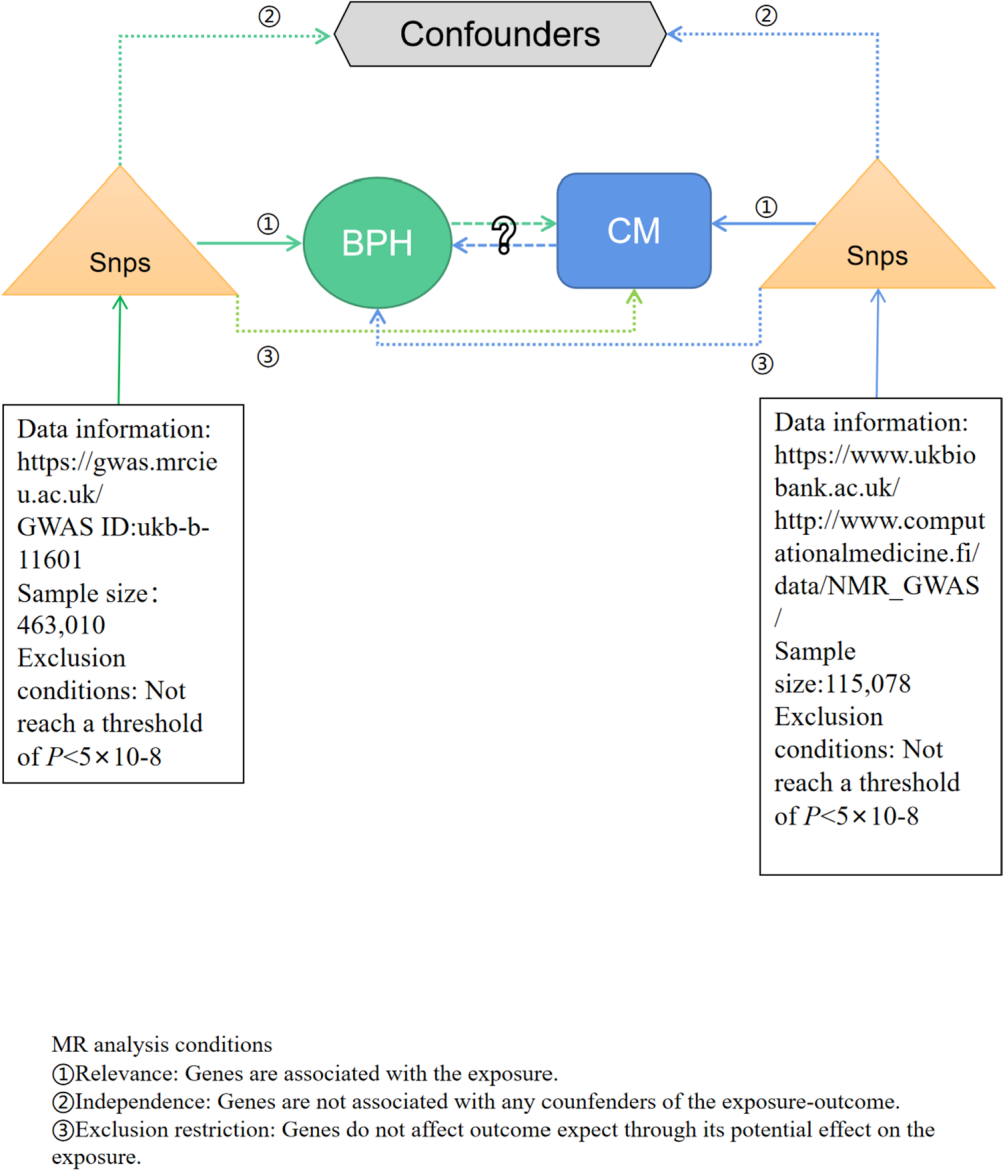
Flow chart

Figure 1 outlines the key steps of the bidirectional Mendelian randomization (MR) study investigating the interplay between CM and BPH. This study involves two MR analyses, using summary statistical data from genome-wide association studies (GWAS), to unveil potential associations between CM and BPH. In the forward MR analysis, CM is treated as the exposure variable and BPH as the outcome. In the reverse MR analysis, BPH is considered the exposure variable and CM the outcome. The fundamental MR assumptions underpinning the study are illustrated in Fig. 1. Given that this study relies on publicly available data, ethical approval is not required.

### MR tool variable selection

The instrumental variables (IVs) for MR analysis were extracted from two distinct GWAS summary results. Initially, a genome-wide significance threshold (*P* < 5 × 10^−8^) was employed [11]. Subsequently, SNP independence was evaluated based on pairwise linkage disequilibrium. SNPs demonstrating an *r*^2^ > 0.001 within a 10,000 kb window were excluded, effectively addressing associations with multiple SNPs and SNPs exhibiting higher *P* values [12]. Linkage disequilibrium signifies the non-random association between alleles at distinct loci. In essence, if two genes are not inherited independently, some level of linkage would be evident. The parameter *r*^2^ ranges from 0 to 1, where *r*^2^ = 1 signifies complete linkage disequilibrium, and *r*^2^ = 0 denotes complete linkage equilibrium, indicating random allocation of the two SNPs. The linkage disequilibrium region's length is denoted by ‘kb’. The threshold *r*^2^ = 0.001 and a 10,000 kb window are used to exclude SNPs with *r*^2^ exceeding 0.001 within 10,000 kb. Additionally, *F*-statistics were computed to gauge the strength of individual SNPs. SNPs with *F*-statistic values exceeding 10 were deemed sufficiently robust to mitigate potential bias.

### BPH data source and tool variable selection

The BPH data originates from the MRC IEU UK Biobank GWAS pipeline version 2 (available at: https://data.bris.ac.uk/data/dataset/pnoat8cxo0u52p6ynfaekeigi). This dataset encompasses a population primarily diagnosed with BPH. The GWAS data is leveraged to identify SNPs associated with BPH, which are subsequently selected as IVs (refer to Supplementary Table 1).

### CM data source and tool variable selection

A summary-level GWAS dataset encompassing 249 CM was acquired from the UK Biobank (unpublished data, accessible via the MRC IEU OpenGWAS database) (see Supplementary Table 1 for details).

Table 1 Detailed information of included data sources

**Table 1:** Detailed information of included data sources

Traits	Sample size	Year	Population	Web source
249 circulating metabolites	115,078	2020	European	https://www.ukbiobank.ac.uk/
Hyperplasia of the prostate	463,010	2018	European	https://gwas.mrcieu.ac.uk/

### MR statistical analysis

SNPs pertaining to both CM and BPH were employed in subsequent forward and reverse MR analyses. The random-effects inverse variance weighting (IVW) method, encompassing the core MR assumptions, constituted the primary statistical approach for estimating potential bidirectional causal associations between CM and BPH [13]. When multiple IVs are available, the IVW method stands as the most robust, as it accounts for variant specificity and causal estimation heterogeneity. The IVW method further encompasses sensitivity analyses, including simple mode, weighted mode, weighted median, and MR Egger regression, to assess research findings' robustness [14]. Should IVs influence outcomes through alternate pathways, indicative of potential pleiotropy, the causal estimation via IVW might incur bias. To assess pleiotropy, MR Egger was utilized. A P-value exceeding 0.05 in MR Egger signifies the absence of level pleiotropy. Heterogeneity testing using MR heterogeneity was conducted to identify SNP-induced heterogeneity. Should heterogeneity be detected, a random-effects model was employed; otherwise, the fixed-effects model was assumed by default. Single SNPs were sequentially eliminated from MR analyses to assess their collective impact [15]. The Twosamplemr (v.0.5.6) within the R package (v.4.3.0) facilitated major statistical analysis and graphical representation [16]. Odds ratio (OR) and the accompanying 95% confidence interval (CI) gauged the extent of risk alteration for each additional standard deviation of exposure factors. Statistical significance was set at *P* < 0.05 [17].

## Results

### Forward MR

**Fig. 2:**
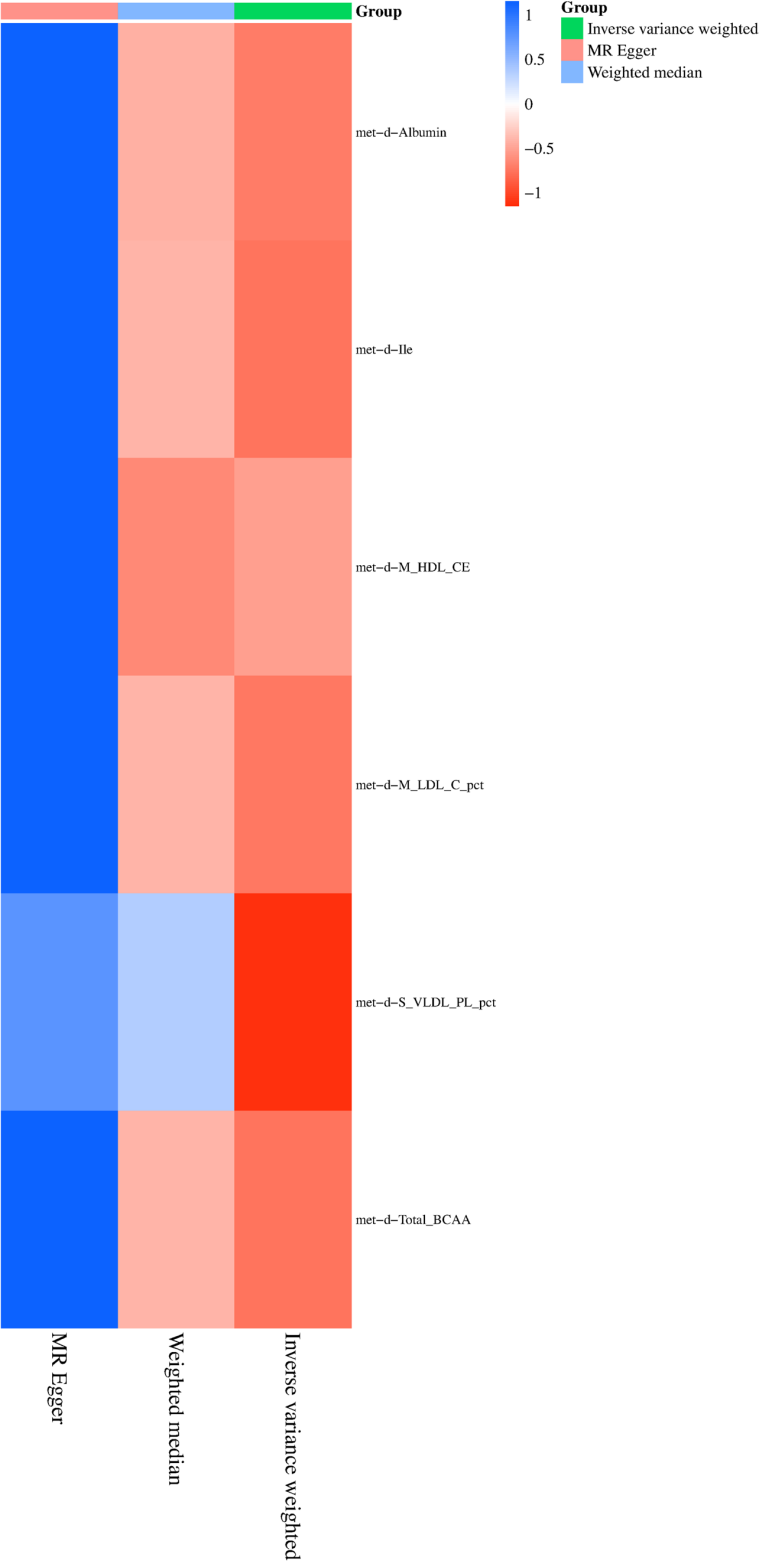
Heatmap illustrating significant correlations in forward MR. The figure showcases varying *P* values in distinct blocks, color-coded from red to blue denoting ascending *P* values. The *X*-axis delineates three separate outcomes: MR Egger, weighted median, and inverse variance weighted. The *Y*-axis signifies different circulating metabolites

**Fig. 3:**
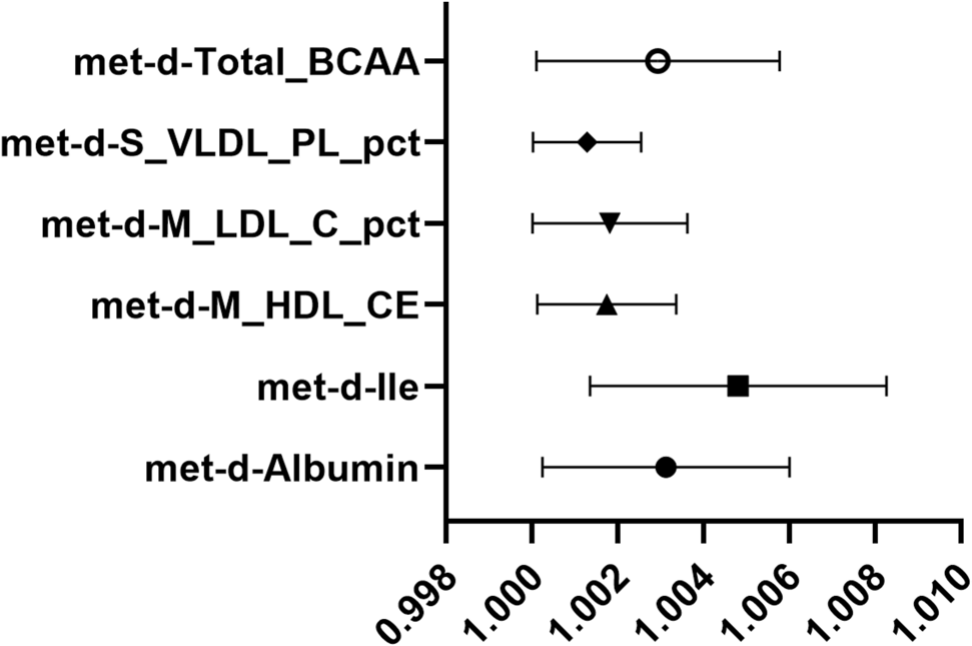
Forest plot depicting significant correlations in forward MR. The *Y*-axis represents diverse circulating metabolites, while the *X*-axis indicates OR values and corresponding 95% CIs. Various shapes in the graph symbolize distinct metabolite ORs, with the horizontal line denoting the range of the 95% CI

The IVW analysis reveals a significant genetic correlation (*P* < 0.05) between six CM and BPH (Table 2, Figs. 2, 3). No substantial evidence of horizontal pleiotropy among SNPs is observed (Table 3, *P* > 0.05). Combining IVW and MR Egger outcomes, no substantial heterogeneity is detected in relation to the association (Table 4, all Cochran’s *Q* *P* values > 0.05).

**Table 2:** Forward MR IVW

id.exposure	id.outcome	Method	nsnp	pval	OR
met-d-Albumin	ukb-b-11601	Inverse variance weighted	20	0.033422337	1.003122226
met-d-Ile	ukb-b-11601	Inverse variance weighted	8	0.006266149	1.004807797
met-d-M_HDL_CE	ukb-b-11601	Inverse variance weighted	65	0.035035974	1.001742073
met-d-M_LDL_C_pct	ukb-b-11601	Inverse variance weighted	25	0.048287504	1.001816397
met-d-S_VLDL_PL_pct	ukb-b-11601	Inverse variance weighted	48	0.045164251	1.001286337
met-d-Total_BCAA	ukb-b-11601	Inverse variance weighted	11	0.041977526	1.002937156

**Table 3:** Forward MR horizontal pleiotropy

id.exposure	id.outcome	Egger_intercept	se	pval
met-d-Albumin	ukb-b-11601	7.17E−05	0.000180985	0.69674802
met-d-Ile	ukb-b-11601	− 4.64E−05	0.000274047	0.871041401
met-d-M_HDL_CE	ukb-b-11601	1.34E−05	7.40E−05	0.856430647
met-d-M_LDL_C_pct	ukb-b-11601	− 6.57E−05	0.000100357	0.518958698
met-d-S_VLDL_PL_pct	ukb-b-11601	6.81E−05	6.44E−05	0.296009917
met-d-Total_BCAA	ukb-b-11601	6.72E−05	0.000153449	0.671559352

**Table 4:** Forward MR heterogeneity

id.exposure	id.outcome	Method	Q_pval
met-d-Albumin	ukb-b-11601	MR Egger	0.4594762
met-d-Albumin	ukb-b-11601	Inverse variance weighted	0.515842519
met-d-Ile	ukb-b-11601	MR Egger	0.702787635
met-d-Ile	ukb-b-11601	Inverse variance weighted	0.798505484
met-d-M_HDL_CE	ukb-b-11601	MR Egger	0.08122939
met-d-M_HDL_CE	ukb-b-11601	Inverse variance weighted	0.094389988
met-d-M_LDL_C_pct	ukb-b-11601	MR Egger	0.540270345
met-d-M_LDL_C_pct	ukb-b-11601	Inverse variance weighted	0.573384125
met-d-S_VLDL_PL_pct	ukb-b-11601	MR Egger	0.921999121
met-d-S_VLDL_PL_pct	ukb-b-11601	Inverse variance weighted	0.916931077
met-d-Total_BCAA	ukb-b-11601	MR Egger	0.855183193
met-d-Total_BCAA	ukb-b-11601	Inverse variance weighted	0.894737334

### Reverse MR

**Fig. 4:**
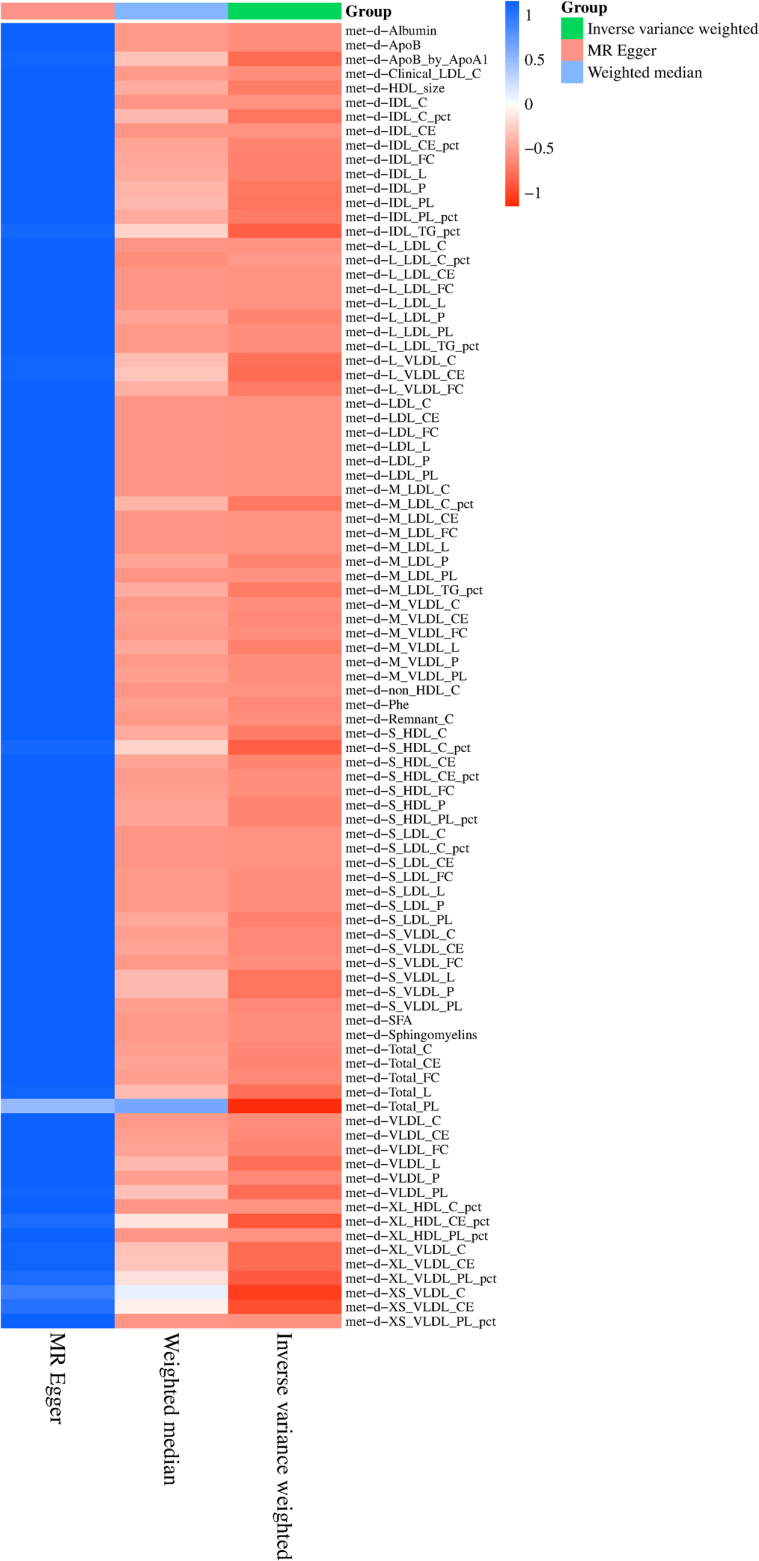
Heatmap displaying significant correlations in reverse MR. The illustration employs distinct colors to signify varying *P* values within individual blocks, transitioning from red to blue to denote ascending *P* values. The X-axis encompasses three distinct outcomes: MR Egger, weighted median, and inverse variance weighted. The *Y*-axis delineates diverse circulating metabolites

**Fig. 5:**
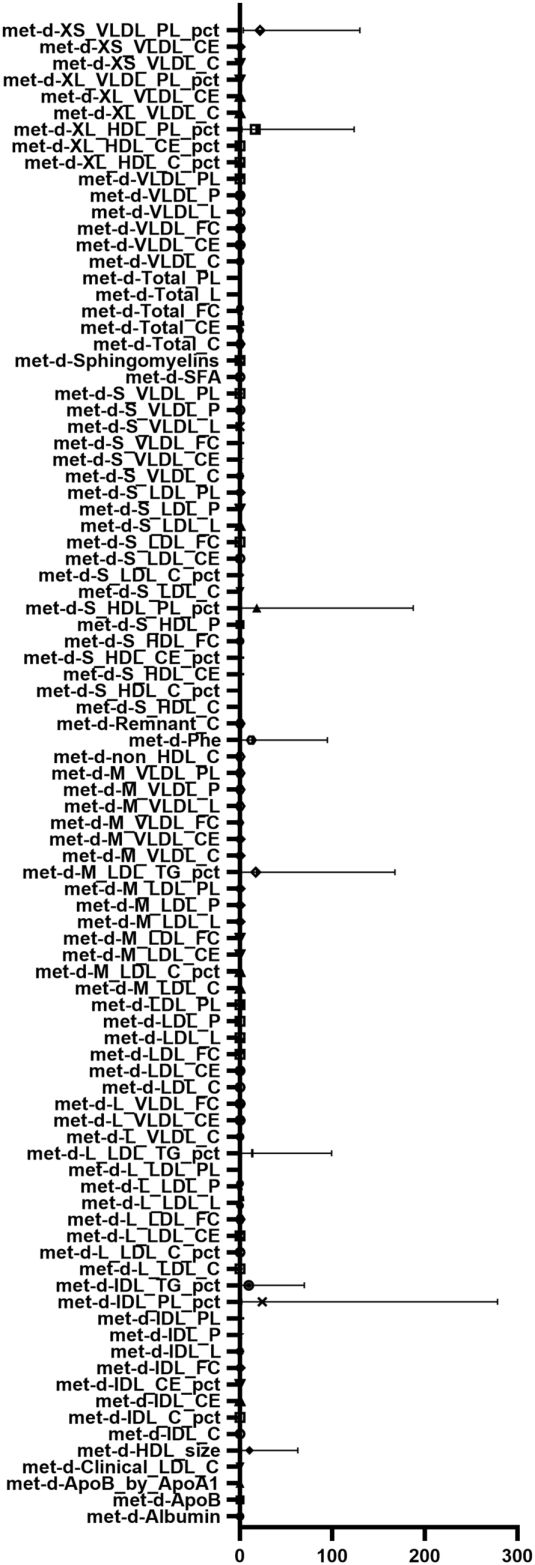
Forest plot illustrating significant correlations in reverse MR. The *Y*-axis represents distinct circulating metabolites, whereas the *X*-axis denotes OR values along with corresponding 95% CIs. Distinct shapes within the graph symbolize distinct metabolite ORs, with the horizontal line marking the range of the 95% CI

The reverse MR analysis demonstrates disparities in 92 metabolites within BPH patients, accounting for heterogeneity and pleiotropy. As exposure escalates, the risk associated with certain metabolites increases, including the Phospholipids to total lipids ratio in IDL, Phospholipids to total lipids ratio in very small VLDL, Phospholipids to total lipids ratio in small HDL, Triglycerides to total lipids ratio in medium LDL, Phospholipids to total lipids ratio in very large HDL, Triglycerides to total lipids ratio in large LDL, Phenylalanine, Average diameter for HDL particles, and Triglycerides to total lipids ratio in IDL. Conversely, the risk of 83 other metabolites diminishes as exposure intensifies (refer to Figs. 4, 5 and Supplementary Table 2).

## Discussion

Benign prostatic hyperplasia (BPH) stands as a prevalent health concern among males, influenced by a complex interplay of biological and environmental factors. The central objective of this study was to investigate the interrelation between circulating metabolites (CM) and BPH via a bidirectional Mendelian randomization (MR) analysis, thereby unveiling potential pathogenic mechanisms. This bidirectional MR investigation has unearthed a discernible association between BPH and CM.

The forward MR analysis divulges six metabolites within the CM realm that manifest associations with BPH, while meticulously addressing issues of heterogeneity and pleiotropy. This lends credence to the genetic impact of these metabolites upon BPH. Notably, elevated exposure to albumin, isoleucine, esters in medium HDL, cholesterol to total lipids ratio in medium LDL, phospholipids to total lipids ratio in small VLDL, and total concentration of branched-chain amino acids (leucine + isoleucine + valine) escalates the risk of BPH, thereby underscoring their potential as pathogenic factors for BPH. The prominence of lipids, amino acids, and albumin in providing requisite nutrients for BPH cell proliferation carries implications for BPH prevention, such as dietary lipid reduction. A study involving 307 men indicated that dyslipidemia and obesity act as BPH risk factors [18]. Obesity, a facet of the metabolic syndrome, is correlated with prostate volume, with the larger prostate size and heightened symptoms often observed among obese individuals [19].

Conversely, the reverse MR analysis highlights disparities in 92 metabolites among BPH patients, possibly signifying biological alterations within the bodies of these patients and reinforcing the nexus between metabolic dysregulation and BPH. These insights hold potential to identify novel avenues and strategies for diagnosing and treating metabolic ailments in the future. Of note, this reverse MR scrutiny underscores that nine key metabolic entities, chiefly phospholipids and triglycerides, heighten the risk in BPH patients, a discovery that could potentially evolve into a supplementary diagnostic criterion for BPH. An animal study has previously suggested a correlation between BPH and elevated triglyceride levels [20]. Additionally, a retrospective clinical study found a significant rise in prostate volume and prostate-specific antigen levels when triglyceride levels exceeded 150 mg/dl [21].

The findings of this study underscore that dyslipidemia constitutes a risk factor for BPH, potentially mediated through genetic influences. Lipids and triglycerides, among other factors, could be genetically intertwined with BPH progression. While the study's findings provide initial insights into the interplay between CM and BPH, a comprehensive understanding of the underlying mechanisms necessitates further investigation. CM might impact BPH development through various pathways including inflammation and lipid metabolism. In the realm of clinical practice, these findings could offer novel perspectives for BPH prevention and treatment, with the potential utilization of CM as biomarkers to assess patient risk and decipher associated biological mechanisms.

Distinct from conventional observational studies, the chief strength of this inquiry lies in its MR-derived causal estimation, effectively circumventing reverse causality and confounding bias. Furthermore, the utilization of extensive and robust GWAS data enhances the precision of outcomes. However, certain limitations intrinsic to this research cannot be disregarded. Notably, the generalizability of outcomes may be constrained due to the predominantly European population used in MR analysis. Subsequent research endeavors could be enriched through larger sample sizes and comprehensive metabolomic analyses, enabling a more nuanced assessment of CM's role in BPH development. As our comprehension matures, the elucidation of the specific mechanisms underpinning CM's action on BPH remains a pertinent avenue for exploration.

### Supplementary Information

Below is the link to the electronic supplementary material.

Supplementary file1 (XLSX 543 KB)

Supplementary file2 (XLS 56 KB)

## Data Availability

Upon request, data can be obtained from supplementary documents or corresponding authors.
